# Experiences and perspectives regarding human papillomavirus self-sampling in sub-Saharan Africa: A systematic review of qualitative evidence

**DOI:** 10.1016/j.heliyon.2024.e32926

**Published:** 2024-06-18

**Authors:** Mathias Dzobo, Tafadzwa Dzinamarira, Ziningi Jaya, Kabelo Kgarosi, Tivani Mashamba-Thompson

**Affiliations:** a School of Health Systems and Public Health, Faculty of Health Sciences, University of Pretoria, Pretoria, South Africa; b Centre for International Programmes Zimbabwe Trust, Harare, Zimbabwe; c Department of Library Services, Faculty of Health Sciences, University of Pretoria, Pretoria, South Africa

**Keywords:** HPV, Self-sampling, Cervical cancer, Screening, Qualitative research, Sub-saharan africa

## Abstract

**Introduction:**

Cervical cancer screening coverage remains low in sub-Saharan Africa (SSA) due to limited access and low uptake of available services by women. The use of HPV-based self-sampling intervention for cervical cancer screening has the potential to increase screening coverage in the region. This study aimed to analyse qualitative evidence on the experiences and perspectives of women, healthcare workers, and policymakers regarding HPV self-sampling.

**Methods:**

We reviewed qualitative studies from January 2011 to March 2023 in PubMed, Scopus, Medline Ovid, Cochrane, and WEB of Science databases for articles with qualitative data on HPV self-sampling from different countries in SSA. The socio-ecological model was used to guide data analysis and the study findings.

**Results:**

Thirteen qualitative studies were included for analysis, and they revealed themes under the intrapersonal, interpersonal, community, and health systems constructs of the Socio-ecological model. Intrapersonal themes included the acceptability of self-sampling, self-efficacy, and the perceived value of self-sampling. The interpersonal construct had themes such as women's spousal relationships, peer support, and the health worker's relationship with the women. The community construct had two themes: social stigma and misinformation, and the influence of cultural norms and religion. Finally, the health systems construct had themes such as the setting for self-sampling, follow-up availability of treatment services and education and awareness.

**Conclusion:**

This study highlights the factors influencing the acceptability and uptake of an HPV-based self-sampling intervention for cervical cancer screening in SSA. Considering these findings when designing interventions in SSA is crucial to ensure acceptance and demand among end-users. Self-sampling interventions offer the potential to reach many unscreened women and increase cervical cancer screening coverage in SSA, which is an essential strategy towards achieving the World Health Organisation's cervical cancer elimination targets by the close of the century.

## 1 Introduction

Cervical cancer is a major global health problem. It is the fourth leading cause of cancer deaths among women worldwide. According to the 2020 Global Cancer Observatory report, more than 600,000 new cases and 340,000 global deaths were recorded in 2020 [1]. The global burden of cervical cancer is unequally distributed as 9 out of 10 cervical cancer cases are reported in low to middle-income countries (LMICs), and 6 out of those cases are found in sub-Saharan Africa (SSA) alone [1].

Due to effective screening and treatment of precancerous lesions and cervical cancer, high-income countries have seen marked decreases in cervical cancer incidence and mortality in recent decades compared to LMICs [2]. These inequalities in cervical cancer prevention are largely due to limited infrastructure, technical expertise and financial resources to initiate and maintain screening programmes in resource-limited settings [3]. The low uptake of cervical cancer screening services by women in SSA is also due to sociocultural barriers associated with interpersonal, social, community and structural factors [4]. Several studies have reported on the barriers that prevent women from accessing cervical cancer screening services in SSA [5,6].

The World Health Organisation (WHO) advocates for human papillomavirus (HPV) testing as an alternative to cytology-based screening in LMICs. Evidence shows that HPV testing is more sensitive than cytology and visual inspection methods in detecting cervical precancer or worse [7,8]. Women have the option of collecting their specimens for HPV testing in a process called HPV self-sampling as opposed to having a health worker collect the specimen [9]. Self-sampling is an acceptable screening option for women in low-resource settings and a key factor in participation and uptake of cervical screening programs in underserved populations [10]. Studies have revealed that HPV self-sampling obviates embarrassment, pain and discomfort associated with a speculum examination by healthcare providers. Additionally, it promotes women's involvement in their sexual and reproductive health affairs [7,11].

Incorporating HPV self-sampling into national screening programmes requires the acceptance and demand for intervention from key stakeholders, including women [12]. This is key for many countries to reach the WHO-set global targets of cervical cancer elimination by the end of the century [13]. To attain these targets, acceptable, easy-to-use, and sustainable screening methods in SSA must be designed. Self-sampling is an innovative tool that can increase coverage by increasing access to hard-to-reach women and screening non-attendees. It is crucial to employ a multi-stakeholder approach in designing an HPV-based self-sampling intervention for cervical cancer screening. In their systematic review in 2021, Camara et al. recommended research involving key opinion leaders and policymakers before implementing a self-sampling intervention [12]. Understanding the experiences and perspectives of all the relevant stakeholders may reveal factors that drive or deter HPV self-sampling at the individual, interpersonal, community and health systems levels.

This review aims to synthesise qualitative evidence on the factors that drive or deter the uptake of HPV self-sampling cervical cancer screening. Several primary studies have addressed the barriers and facilitators to HPV self-sampling in SSA, however, this review aims to address the gap in synthesised literature evidence on the experiences and perspectives of key stakeholders regarding HPV self-sampling. Considering the SSA region has unique socio-cultural practices and healthcare systems, it is crucial to understand the experiences and perspectives of stakeholders to inform practice and future research. Qualitative research is crucial for understanding individual perspectives and experiences that cannot be measured through numerical data or statistical procedures. We anticipate our findings will be useful to policymakers within cervical cancer prevention and control programmes in informing the design of context-specific HPV self-sampling interventions that encourage women to undergo screening, ultimately reducing the incidence of cervical cancer in the region.

## 2 Methods

### 2.1 Protocol and registration

The methods for this systematic literature search have been developed according to the recommendations from the Preferred Reporting Items for Systematic Reviews and Meta-Analyses (PRISMA) statements [14]. The systematic review will follow the protocol that was submitted and registered on PROSPERO (registration number CRD42022377297.

### 2.2 Design

This systematic review builds on the findings of a scoping review conducted to establish the acceptability of HPV self-sampling for screening cervical cancer in SSA. The scoping review was published elsewhere [15]. A systematic search to identify literature on the acceptability of self-sampling for HPV testing was undertaken across 5 major electronic databases and a grey literature search. This systematic review followed the same search strategy [15] and considered qualitative and mixed methods studies that showed evidence of the experiences and perspectives of women, health workers and policymakers regarding self-sampling for HPV-based cervical cancer screening. Studies were included if they employed qualitative research designs such as focus groups, in-depth interviews and semi-structured interviews. To determine the eligibility of the research question, the participant, exposure, and outcome (PEO) nomenclature (Table 1) was followed.

**Table 1:** The PEO nomenclature.

Criteria	Determinants
Participant	women, health workers, policymakers
Exposure	HPV self-sampling intervention (actual or hypothetical) conducted in sub-Saharan Africa
Outcome	experiences and perspectives regarding HPV self-sampling

### 2.3 Identifying the research questions

The research question is: What is the evidence on the experiences and perspectives of women, health workers and policymakers regarding HPV self-sampling in SSA?

### 2.4 Search strategy

We conducted a comprehensive literature search of relevant articles from PubMed; Scopus, Medline Ovid, Cochrane, and WEB of Science electronic databases. We limited the dates of publication from January 2011 to March 2023. We chose to include studies in this period because HPV testing in Africa started gaining traction in response to the WHO endorsement of the use of HPV tests for cervical cancer screening [16]. The first author developed the literature search with the University of Pretoria (UP) librarian (KK). We included studies that reported evidence on women, health workers, and policymakers ‘experiences and perspectives regarding self-sampling for HPV-based cervical cancer screening in SSA. Review articles (narrative, scoping, systematic, meta-analysis, and meta-synthesis) were excluded. The database search terms included “cervical cancer”, “human papillomavirus”, “self-sampling”, and “sub-Saharan Africa”. Boolean terms, AND and OR, were used to separate the keywords. The keyword search also included medical subject headings (MeSH) terms. The search strategy was adapted to suit each database. In addition, we also searched the WHO library and university repositories for grey literature such as dissertations, theses, and reports. Following keyword search, eligible studies were exported to the EndNote version 20 library for abstract and full article screening. The EndNote library “Find full text” option was used to download the full texts of exported studies**.**

## 3 Study selection and inclusion criteria

### 3.1 Study selection

The principal investigator screened titles using the eligibility criteria as a guide. Eligible articles were exported to EndNote 20 library, where duplicates were identified and removed. MD and ZJ then independently screened the abstracts to identify studies for full-text screening with guidance from the eligibility criteria for this study. Following the abstract screening, two authors, MD and ZJ, reviewed full texts for eligibility using a pretested screening instrument. Discrepancies in screening decisions between reviewers were resolved through discussion and consensus; a third reviewer, TD, was consulted when necessary. The reference lists of included studies were also searched for relevant literature, and where full texts were difficult to access, the authors were conducted through email. Qualitative studies were selected based on their thematic focus. We thoroughly read a study's objectives, methodology, and findings to look for recurring themes. We examined a study's findings to see if they aligned with our research question. A study was included if its main findings addressed stakeholders' experiences and perspectives on HPV self-sampling in SSA.

#### 3.1.1 Inclusion criteria

• Qualitative studies, i.e. interviews, focus group discussions, surveys or questionnaires with open-ended questions and qualitative components of mixed methods that show evidence of experiences and perspectives regarding self-sampling.
• Studies involving women, health workers and policymakers
• Studies conducted in SSA
• Studies conducted between January 2011 to March 2023

#### 3.1.2 Exclusion criteria

• Quantitative studies and quantitative components of mixed methods studies
• Stakeholders other than the ones mentioned in the inclusion criteria
• Studies in any geographical location other than SSA
• Studies published before January 2011

### 3.2 Theoretical Framework

This study will be guided by the Socio-ecologic model (SEM) [17]. In this study, the SEM conceptualises an HPV self-sampling screening intervention broadly, emphasising the interplay of different factors on its acceptability for cervical cancer screening in SSA. The SEM was first suggested by Broffenheimer in the 1970s [18] and later redefined by McLeroy et al. [19]. The SEM typically includes 5 levels of influence: intrapersonal, interpersonal, healthcare, community and health systems [17].

### 3.3 Assessment of methodological quality of included studies

All included studies were critically appraised using the Critical Appraisal Skills Programme tool or CASP for qualitative research [20]. Two independent reviewers (MD and ZJ) conducted the appraisal exercise. The tool has ten questions, each focusing on a different methodological aspect of a qualitative study. The questions posed by the tool ask the researcher to consider whether the research methods were appropriate and whether the findings are well-presented and meaningful. Each question was scored using the ' Y′, ‘N’ or ‘Can't tell’ answer keys.

## 4 Data extraction and analysis

### 4.1 Data extraction

A Microsoft Excel data extraction form was developed by the principal investigator to include the following study characteristics: authors, publication date, study design, research aim, country, study setting (rural/urban), self-sampling intervention (actual/hypothetical), method of data collection, participants (women, health workers, policymakers), main outcomes. Two independent reviewers (MD and ZJ) extracted the data from all the included studies. Any disagreements that arose from the extraction process were discussed until a consensus was reached. Where necessary, a third reviewer (TD) resolved the discrepancy.

### 4.2 Data synthesis

The thematic synthesis approach was used to pool qualitative findings from this study. The approach developed by Thomas and Harden [20] specifically looks at individual perspectives and experiences using an integrative approach that considers data from comparable primary studies. We used NVivo 13.0 for data coding. Generated codes were identified and grouped into themes. The thematic synthesis approach was performed in three stages.

• **Coding text**: each study was coded line-by-line, extracting data that answers the research question (this was conducted by the principal investigator)
• **Developing descriptive themes**: the codes identified in the first stage were categorised based on similarities to create themes (this was conducted by the principal investigator)
• **Generating analytical themes**: the themes identified in the second stage were used to develop key messages

The outcome of the coding process was verified and discussed with TMT, a senior researcher and principal investigator. The process of cross-checking the outcome of coding involved a thorough discussion of the key components of each included article, such as the study aim, setting, number of participants, data analysis method, main findings (themes), limitations, and conclusions. The findings of the study were reported using the Preferred Reporting Items for Systematic Reviews and Meta-Analyses (PRISMA) checklist to ensure transparency [14].

## 5 Results

### 5.1 Screening results

**Fig. 1:**
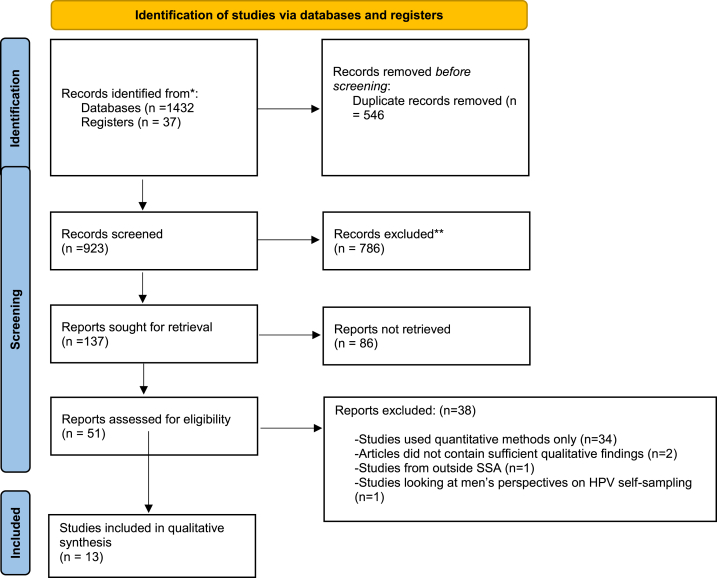
Preferred Reporting Items for Systematic Reviews and Meta-Analyses flow diagram of the study selection process.

The electronic databases and searches from other sources identified 1432 and 37 articles respectively (Fig. 1). These were exported to EndNote 20 library. The results retrieved from each database are displayed in Supplementary File 1. After removing duplicates, a total of 923 records remained. Titles and abstracts of these remaining records were screened and eliminated based on the exclusion criteria. A total of 137 articles were removed at the abstract stage because they formed part of the exclusion criteria. Fifty-one studies remained after abstract screening and were eligible for full article screening. Thirty-eight articles were excluded at the full article screening stage, 33 articles [7,11,[21], [22], [23], [24], [25], [26], [27], [28], [29], [30], [31], [32], [33], [34], [35], [36], [37], [38], [39], [40], [41], [42], [43], [44], [45], [46], [47], [48], [49], [50], [51], [52]] had quantitative study designs and 2 studies [53,54] did not provide sufficient qualitative evidence on the experiences and perspectives regarding HPV self-sampling, 1 study included women from outside SSA region [55] and 1 study had men's perspectives on HPV self-sampling only [56]. The remaining 13 articles were included in the qualitative synthesis [[57], [58], [59], [60], [61], [62], [63], [64], [65], [66], [67], [68], [69]].

### 5.2 Characteristics of included studies

Thirteen studies [[57], [58], [59], [60], [61], [62], [63], [64], [65], [66], [67], [68], [69]] were included for qualitative synthesis (Table 2). Of the 13 studies, 10 had qualitative and 3 had mixed methods designs. All the included studies were conducted between 2014 and 2021. Nine African countries were represented in the included studies: Uganda [58,59,64], Kenya [62,65], Ethiopia [57,60], Cote d’Ivoire [67], Cameroon [66], Ghana [61], Malawi [63], Tanzania [69] and South Africa [68].

**Table 2:** Characteristics of included studies.

Author Year	Country	Study Aim	Participants	Sample size	Setting (Urban/Rural)	Setting of self-sampling	Method of data collection	Key findings-
Saidu et al., 2019 [68]	South Africa	To explore women's perceptions and acceptance of self-collection of samples for cervical screening and their willingness to do so, in a low-resource setting in South Africa	Women	41	Urban	Health facility	Focus group discussion	Women's attitudes towards self-sampling were positive. The lack of confidence to collect a good specimen was reported and generally, women would prefer a sampling strategy that cut travelling costs.
Bakiewicz et al., 2020 [69]	Tanzania	To investigate the feasibility and acceptability of HPV self-sampling among Tanzanian women who attended a patient-initiated cervical cancer screening compared to provider-based HPV sampling	Women	21	Urban	Health facility	Semi-structured interview	Home sampling was most preferred but due to the lack of confidence, the presence of a health provider was needed. There is a need for additional research on preferred sampling devices
Lee et al., 2021 [63]	Malawi	To explore screen-and-treat experience, acceptability of the program and attitudes towards self-sampling for HPV testing as an alternative screening method	Women	17	rural	community/villages	semi-structured interview	Screen and treat strategy with thermocoagulation was well accepted by women. Reaching out to men presents a unique opportunity to increase the acceptability of self-sampling, particularly among rural women
Oketch et al., 2019 [62]	Kenya	To determine women's perspectives and experience with HPV self-sampling	Women	10	Rural	Community and health facilities	In-depth interview	Understanding barriers and facilitators of self-sampling is key to designing programmes that are acceptable to women. Easy access, privacy and convenience are facilitators of self-sampling. Raising awareness may increase the uptake of self-sampling
Behnke et al., 2020 [61]	Ghana	To explore female HCPs' perceptions, advocacy for, and implications of self-sampling to enhance self-sampling acceptability in the targeted screening population	Healthcare workers	10	Rural	Health facility	Semi-structured interview	Health worker experience of self-sampling is important for future implementation of a self-sampling programme. Health workers can advocate for self-sampling
Brandt et al., 2019 [60]	Ethiopia	To explore perceptions and acceptability of HPV self-sampling-based cervical cancer screening among community members and health professionals in rural northwest Ethiopia and to identify preferences and socio-cultural barriers regarding self-sampling	Healthcare workers and Women	Healthcare workers = 4 Women = 41	Rural	Health facility	Focus group discussion = 41 key informant interviews = 4	Women preferred home-based self-sampling and raising awareness and involving family support were identified as facilitators of self-sampling, religion cultural leaders and male involvement may help to increase coverage of screening using self-sampling
Bansil et al., 2014 [59]	Uganda	To determine women's and providers' experiences with self-sampling, women's opinions of sampling at home, and their future needs	Healthcare workers and Women	Healthcare workers = 52 Women = 20	urban	health facility	Semi-structured interviews	HPV self-sampling is acceptable to health providers and they can play an important role in improving women's proficiency in performing self-sampling.
Roux et al., 2021 [66]	Cameroon	To explore potential barriers to human papillomavirus (HPV)-based cervical cancer screening from a healthcare provider (HCP) perspective in a low-income context. Second, we aimed to explore the acceptability of a single-visit approach using HPV self-sampling.	Healthcare workers	16	Rural	Not performed	Focus group discussion	Understanding healthcare workers' perspectives on the underutilisation of available screening services is of importance considering the pivotal role that they play within a cervical cancer screening programme. Self-sampling is seen as a tool that empowers women to be involved in their health
Rawat et al., 2020 [58]	Uganda	To understand the knowledge, preferences, and barriers for self-collected cervical cancer screening (SC-CCS) and follow-up care at the individual and health system level to inform the implementation of community-based SC-CCS.	Healthcare workers and Women	Healthcare workers = 13 Women = 45	Rural	Not performed	Focus group discussion	Women preferred self-sampling at home but stated need follow-up and treatment at the health facility. Integration of HIV and cervical cancer programmes was seen as a potential solution to increase health services access to women. Empowering women with knowledge of the benefits of self-sampling was perceived as a facilitator to women taking up self-sampling in the future.
Mensah et al., 2020 [67]	Côte d’Ivoire	To assess the acceptability of HPV screening among HIV-infected women before the implementation of this method to adapt it to the societal context in Abidjan	Women living with HIV	21	Urban	Not performed	In-depth interview	Healthcare givers within HIV programmes have a role to play in cervical cancer screening. The trust that exists with patients and health workers within an HIV programme can facilitate uptake in cervical cancer screening programmes.
Megersa, 2020 [57]	Ethiopia	To explore the knowledge, perceptions, and beliefs towards cervical cancer, screening and the barriers to the acceptance of self-sampling in society	Women	47	Rural	Community/home sampling	In-depth interview = 22 focus group discussion = 25	Women reported barriers to home self-sampling such as husband disapproval, social influence and lack of knowledge on cervical cancer and HPV self-sampling. Both women and their husbands were misinformed about the self-sampling device. Integrating women's health services with cervical cancer screening has the potential to increase screening coverage.
Podolak,2017 [65]	Kenya	To determine how local decision makers could apply a multimethod approach to make strategic decisions to effectively implement a Cervical Self-Sampling Programme in Kenya	Policymakers, men and General population	Policymakers (women = 21, men = 9) -General population (women = 94 men = 3)	Urban	Not perfomed	Formal interviews and focus groups	The stakeholders agreed on three main things namely that there was political will to improve CC screening, but resources to fund were inadequate and also successful implementation of CC screening would need to be subsidised to make it affordable to women and lastly sampling was socially acceptable and its introduction would increase demand for screening and treatment.
Teng, 2014 [64]	Uganda	To (1) define embarrassment and develop an understanding of the role of embarrassment about cervical cancer screening and self-collected HPV DNA testing; (2) determine viable solutions to overcoming barriers to; and (3) better understand embarrassment as a barrier to screening	Healthcare workers and Women	Healthcare workers = 6 Women = 16	Urban	Not performed	Focus group discussion = 16 Key informant interview = 6	psychosocial barriers such as stigma, embarrassment and shame are responsible for the low uptake of screening by women. Understanding these barriers before the design of a programme is key to addressing the barriers and increasing the acceptability and uptake of cervical cancer screening

### 5.3 Quality of studies included

According to the CASP tool results, all the studies (n = 13) had either no or minor methodological limitations (Refer to supplementary file 2 for detailed results). Most studies clearly stated aims and objectives and appropriately used a qualitative methodology. All of the studies collected data in a way that addressed the research question(s) and was suitable for data synthesis.

### 5.4 Main findings

The findings on the experiences and perspectives regarding HPV self-sampling were grouped into the four categories of the SEM: (1) intrapersonal, (2) interpersonal, (3) community and (4) health systems factors.

### 5.5 Intrapersonal factors

Our study findings revealed the following sub-themes under the intrapersonal construct of the SEM: the acceptability of self-sampling, self-efficacy, and the perceived value of self-sampling.

### 5.6 Acceptability of self-sampling

The acceptability of self-sampling was a recurring theme in seven of the studies. Women's reasons for preferring self-sampling included the involvement in their health and being able to collect the specimen without anyone touching them [57,59,[61], [62], [63],^68^,^69^]. An additional motivation for performing self-sampling was the relatively easy and painless way of obtaining a specimen compared to a conventional speculum examination by a clinician [59,62,63,68]. Women also preferred self-collection because they feared contracting diseases from the use of a speculum tool, which they thought was unsterile [69]. The privacy associated with self-sampling was a driver of uptake by women who were naive to self-sampling and those with self-sampling experience [59,62,68]. Privacy was a key driver to women performing self-sampling, particularly among women reluctant to have a specimen collected by a male health worker [62] and women who did not want to meet up with healthcare practitioners who had examined them. This was echoed by a health worker who participated in a workplace self-sampling screening activity:

*"I think this one [self-sampling] is better – because of the Pap smear, I have to come here and lie down for somebody to take the sample. Because there I think the privacy you are shy, you don't want anybody to look at your private part or people you know around, your colleagues, doing it for you. It's better [if] you are in the comfort of your home and take your sample. So that one is better than the first one."* (Health worker, Ghana) [61].

### 5.7 Self-efficacy

Seven studies discussed the self-efficacy of self-sampling [59,60,62,64,[67], [68], [69]]. Despite their preference for self-sampling, some women reported a lack of confidence in performing the procedure and instead trusted a clinician to collect a quality specimen and also identify any other abnormalities within their genital area [59,[67], [68], [69]].

The lack of formal education was reported as one of the reasons for the lack of self-confidence to perform a self-sampling procedure [58,69], resulting in some women needing the presence of a health worker to perform self-sampling correctly. Despite their willingness to assist women with instructions, healthcare workers bemoaned the over-dependence of some women on healthworker assistance even after receiving adequate training and instructions.

*“It wasn’t easy to show the procedure of self-sampling for some of the women; we spent a lot of time to make them understand how to use it (the self-sampling device). Some of them forgot every step of the procedure immediately after they went to their bedrooms to collect the sample,”* (Health Worker, Uganda) [58].

Another reason for the lack of confidence to perform self-sampling was the fear of the safety of the sampling device, especially by women with no experience with the procedure. The health workers were very useful in dispelling any misinformation about the devices and also assured women of the validity of HPV results [59].

### 5.8 Perceived value of self-sampling

This sub-theme highlighted women's perceived benefits of performing self-sampling and the perceived consequences of not screening or delaying cervical cancer screening. This was revealed in three studies [57,62,68]. According to most women, the fear of death due to cervical cancer was the main motivation for seeking and utilising screening services. A participant reported that cervical cancer is a killer disease, and the moment she heard of screening through HPV self-sampling, she decided to go for screening and was ready to receive treatment in case she was HPV positive.

*“I have seen cervical cancer kill those who did not want to go to the hospital for screening. That is why when I heard about it, I decided to go for the screening so that in case I am HPV positive, I find help****”*** (Participant, Kenya) [62].

This study revealed that not all women are aware of the risks associated with cervical cancer, as some may not consider screening necessary if they feel healthy and are asymptomatic. It is crucial to educate and raise awareness among women to ensure that they understand the gravity of the potential consequences of not attending screening early.

### 5.9 Interpersonal factors

Nine studies [57,58,[60], [61], [62], [63], [64],^68^,^69^] reported evidence of the interpersonal factors that influence the acceptability and uptake of HPV self-sampling. In this study, we grouped findings into three sub-themes, namely the social relationships between women and their spouses, peers and health workers.

### 5.10 Women-spouse relationship

In five different studies [57,58,60,62,63], a common sub-theme emerged, which showed that women who received support and encouragement from their spouses were more likely to participate in self-sampling interventions. Women were more likely to participate in a self-sampling intervention if their spouse understood the risks of cervical cancer and encouraged them to seek early screening. However, the need to seek spousal permission before participating in a self-sampling screening intervention was found to be a significant barrier in several studies [57,58,60,62]. According to health workers, gender inequalities within African societies pose a significant barrier to women making decisions about sexual and reproductive health. Women often face physical violence and, in some cases, their partners may leave them after a positive HPV result. This makes it hard for women to communicate positive results to their partners and to seek treatment [58].

*“If they test the woman and she is positive, then their relationship with her husband may perish … If my husband gets to know that I have cancer – shall we remain the same? Instead, she keeps quiet and starts rotting with the disease, not knowing that it is affecting her because she doesn’t want her husband to know that she is infected with cancer in fear of abandoning her.”* (Participant, Uganda) [58].

Inadequate information and awareness on cervical cancer were the cause for the lack of understanding by men and their refusal to permit their spouses to attend cervical cancer screening [60]. Some men associated the act of self-collection with the defilement of their women [57,60].

### 5.11 Women-health worker relationship

The evidence of the relationship between the women and health workers was reported in 6 studies [57,58,62,[67], [68], [69]]. Some of the participants were willing to have their specimens collected by a health worker whom they identified as capable and experienced to perform the procedure correctly [[67], [68], [69]]. Women also trusted the health worker to identify other problems within their genital area during specimen collection, highlighting the dependence of women on health workers [68]. Despite the good relationship between health workers and women, the attitude and behaviour of some health workers were a deterrent to the uptake of self-sampling by some women. A woman from Uganda stated that:

*Some people fail [to go to facilities] because they have been disappointed by the health workers' attitudes, so next time you tell someone to go – one will say 'No, I cannot go there' – simply due to the health workers' bad attitude and behaviour.”* (Participant, Uganda) [58].

### 5.12 Peer support

Two studies reported evidence of the effect of peer encouragement or peer support on the perception and uptake of self-sampling by fellow women [62,64]. There was a willingness to participate in self-sampling when women were encouraged by other women with self-sampling experience. The encouragement was particularly useful to women who were naïve to performing self-sampling and lacked the confidence to conduct the procedure [62,64].

“*Through these people who have self-collected and through more training, they also help those who have never attended the training, who have never self-collected, to make them confident that self-collection is not painful. You do it yourself, it’s not the doctor doing it. I think that through them, more people will come to do the self-collection*” (Participant, Uganda) [64].

### 5.13 Community factors

At the community level of the SEM, our study revealed stigma, misinformation, cultural and religious practices as factors that affect women's acceptability and uptake of HPV self-sampling. The evidence of community factors on women's acceptability of self-sampling was discussed in 4 studies [57,58,62,64]. Among the barriers that women faced in accessing cervical cancer screening services was stigma. Health workers in Uganda reported that women were afraid of getting home visits by healthcare providers as this would be associated with having HIV [58]. In Kenya, women feared that engaging in or participating in cervical cancer screening would be seen as having cervical cancer disease [62]. Additionally, women were unwilling to participate and give a self-collected specimen to a health worker because of fear of backlash from other members of the community who were against self-sampling [57].

*“Later, some of the residents of our local community insulted me for participating in the screening and everybody was blaming me because I said yes to those girls (sample collectors); I was really embarrassed for giving that sample to those girls”.* (Participant, Ethiopia) [57].

The stigma towards women was attributed to a lack of information, education and awareness about cervical cancer disease and the self-sampling method. In particular, the community had misconceptions about the role of the sampling device, which they suspected could impregnate women, hence the refusal by husbands to participate [57]. Additionally, women believed that the act of self-sampling using a brush was against the doctrine of their religion. In particular, the wives of religious leaders perceived the use of sampling devices as defiling [57]. Another barrier to the uptake of self-sampling was the cultural norm that forbade women from touching themselves in their genital area [59].

### 5.14 Health systems factors

The following studies revealed the health systems factors affecting the acceptability, uptake and implementation of an HPV self-sampling screening programme [57,58,60,[62], [63], [64], [65], [66],^68^,^69^]. The sub-themes that emerged included the setting for self-sampling, linkage to care and education and awareness.

### 5.15 Setting for self-sampling

Opinions on the best place to perform self-sampling varied, but most preferred a health facility to avoid making a trip to collect a sampling kit and another to return the specimen. Women were also afraid of contaminating the specimen at home or forgetting to return it to the facility in time. Another reason for the preference to perform self-sampling at the health facility was the need for assistance from a health worker to ensure the collection of an appropriate specimen.

*“I would wish for the clinician to be there so that after getting tested and I end up being positive the doctor can [perform the] treatment already but not that I look for money and go to Migori”* (Participant, Kenya) [62].

Some women preferred the option of community-based self-sampling for cervical cancer screening. This was because it was more convenient as they did not have to travel long distances to a health facility. Additionally, they could complete the self-sampling procedure at home and return to their daily household chores [62,63]. Privacy and convenience were the main drivers for home-based self-sampling. Since women could perform self-sampling at home, it was believed that this would increase participation rates by overcoming women's inability to travel to a distant health centre.

### 5.16 Linkage to care

A recurrent theme among the included studies was the need for follow-up and linkage to care after a positive HPV result. Women stated the importance of having nearby treatment services as this made them feel secure even after getting a positive HPV test result. The lack of such services was noted as being a barrier to women taking part in self-sampling as they did not want to live in fear of being HPV positive without an option for treatment.

*“If there is no treatment after the examination, people will not want to be screened for cervical cancer.”,* (Participant, Ethiopia) [60].

Some women who participated in HPV self-sampling reported a concern about a delay in receiving notification of a positive HPV result. They mentioned that not knowing the results caused them anxiety and that they felt much calmer when they received the results in time. This allowed them to seek treatment or further medical care without any delay. They also stated that getting treatment on the same day of screening was easier than waiting for the results. Health workers pointed out the challenges of convincing women to repeat self-sampling when past HPV results have not been sent to them on time.

*“Many women did not receive their results and those who identified as having the disease were not linked to follow-up and treatment centers. Currently, we feel shame to meet the community members as we have promised them to bring back their results and to link them to follow-up and treatment center in case, they are diagnosed with the disease during sample collection.”* (Health worker, Ethiopia) [57].

The provision of free cervical cancer screening services at the health facility or designated community points facilitated performing self-sampling. Most of the women in the included studies are from a low socio-economic status background, and some of them receive free HIV services and care in their communities and prefer cervical cancer screening services to be the same. The free services motivated women to convince their peers and raise awareness about cervical cancer screening via self-sampling [61,67].

*“The free access also encouraged me. Because when you arrive and people tell you that you have to pay, you hesitate a little bit. The free access encourages. It is the reason why when I went back home, I told women from my neighborhood to go get screened.”* (Participant, Ghana) [67].

### 5.17 Education and awareness

Six studies revealed evidence of education and awareness of cervical cancer [[57], [58], [59],^62^,^64^,^68^,^69^]. Both women and health workers emphasised the importance of education for alerting women to the risks and the value of knowing their HPV status [58,62,64]. Health workers highlighted the importance of educating family and community members to ensure that women have support from their families, as this has been noted as a barrier to women accessing cervical cancer screening services [60]. The majority of women preferred to receive educational information through verbal communication from trained health workers and through pamphlets. Education was described as a tool that eliminates the fear of performing self-sampling [58]. When asked about the use of diagrams and illustrations for conveying information, women had different views, and most of them thought the diagrams may show inappropriate images [68]. A participant who took part in a focus group discussion in South Africa had this to say:

“*Those pictures of cervix and wombs are scary, I would prefer an explanation and a diagram because the doctor will explain and at the same time show you on the picture how to do it [self-sample].”* (Participant, South Africa) [68].

## 6 Discussion

This systematic review summarises the qualitative evidence on the experiences and perspectives of key stakeholders regarding HPV self-sampling in SSA. Our findings are crucial for future research and practice, and their relevance cannot be overemphasised, given that HPV testing is increasingly being advocated for and adopted as the primary screening method of choice. The study revealed the drivers and deterrents of HPV self-sampling screening method at the SEM's intrapersonal, interpersonal, community, and health systems levels.

At the individual level, women accepted the utility of self-sampling for enabling them to access cervical cancer screening services in a more private, confidential and less embarrassing manner compared to clinician collection or speculum examination. The option to perform self-sampling empowers women and allows them to play an active role in their sexual and reproductive health rather than as mere bystanders. Our results are consistent with findings from a systematic review of randomised control trials comparing the uptake between clinician sampling and self-sampling. The study revealed self-sampling as a more attractive screening option because of the privacy it afforded women [70]. HPV self-sampling additionally helps women to overcome the embarrassment associated with a Pap smear or a pelvic examination by a clinician [71]. Another review by Nelson et al. shows that HPV self-sampling is acceptable for cervical cancer screening as women found it easy, less painful and convenient to collect a vaginal specimen for HPV testing [72].

A common concern both women and health workers raised was the lack of self-efficacy in performing self-sampling. The lack of self-efficacy reverses the anticipated gains of self-sampling-based cervical cancer screening to empower women and increase access to underserved communities. Similar to our findings, Tesfahunei and colleagues revealed that women were willing to perform self-sampling for future screening appointments and were glad to recommend it to family or friends but would prefer to have a clinician collect the specimen due to a lack of confidence to perform the procedure [70]. The lack of adequate education has been cited as the major reason for poor proficiency in self-sampling in SSA. Women who reported their lack of confidence in performing self-sampling had attained a low educational status [7,73]. The reported lack of confidence calls for tailored education to improve women's confidence to perform a self-sampling procedure correctly to achieve a high uptake of HPV screening in SSA.

Despite the positivity around HPV self-sampling, there are concerns over the use of the sampling device for fear of violating cultural norms which forbid women to touch themselves. This is not uncommon, especially in Muslim-dominated societies which value modesty and sexual purity of women. A study conducted in Morocco within a Muslim-dominated community reported that women were reluctant to perform self-sampling for fear of losing their virginity [74]. The fear of defilement presents a challenge to implementing an HPV self-sampling intervention and warrants extensive culture-sensitive education and awareness. Additional safety concerns over the sampling devices were noted. Previous research reports on women's preference for visually appealing devices resembling a cotton swab as they felt more comfortable using a device familiar with a swab they have seen before [75]. The option of urine self-collection warrants exploration as urine is non-invasive, and its utility for HPV detection has been demonstrated [76]. There is limited evidence on the preferences for sampling devices in SSA; further research exploring preferences for different sampling devices and methods is needed to increase the acceptability of self-sampling and willingness to self-sample. Furthermore, women with a positive self-sampling experience are more likely to recommend screening methods to their colleagues and family.

A key finding of our study is the role of the community, fellow women, and spouses in women's acceptance and uptake of self-sampling. Social relationships are a critical part of women's lives, particularly in SSA, and they play a key role in determining acceptance of health interventions. It is important to note the influence that men yield over their spouses to determine if they should seek certain health services. The patriarchal nature of most African societies presents a significant barrier to the provision and access to cervical cancer screening services, particularly in remote and rural areas that are already marginalised and underserved [77,78]. This study expands on the findings by Camara et al., whose review highlighted the important role of social relationships, particularly within male-dominated cultures [12]. To overcome this barrier, there is a need to empower women through education and involve men as key partners in women's sexual and reproductive health programmes. Education can potentially dismiss myths and misinformation by assuring men of the importance of the procedure, confidentiality, and privacy to gain their trust and cooperation. A key facilitator of self-sampling uptake revealed in our study is that of fellow women with self-sampling experience. Lott et al. reported the effectiveness of peer-to-peer education in raising awareness to increase cervical cancer screening uptake, and this model was effective because of existing social ties and familiarity of the peer educators with the community members [79,80]. Involving community women who have previously performed self-sampling during awareness campaigns to give testimonies may increase the acceptability and uptake of the interventions in their communities since women can trust people they know and interact with regularly.

A significant barrier to self-sampling uptake is the role of cultural and religious restrictions, which view the act of women touching themselves as an abomination. It is in the best interest of programme managers to engage the wider community, including traditional and community leaders, to embrace self-sampling as a method that improves women's health and well-being rather than a violation of cultural and religious norms. The stigma associated with a positive result is another barrier that needs to be addressed, as women are generally afraid of communicating a positive result, which is perceived by the community as a result of promiscuity or unsafe sexual practices. The stigma associated with a positive HPV result was reported as a deterrent to participating in cervical cancer screening. Mccaffery et al. reported that women faced psychosocial challenges due to testing HPV positive because it was associated with promiscuity or marital infidelity [81]. Additional qualitative research on the psychosocial impact of a positive HPV result in SSA is needed to gain a deep insight into the impact of a positive HPV result on women's mental well-being.

The study received mixed opinions regarding the ideal location for self-sampling. Some women preferred to take the self-sampling test at a healthcare facility because they lacked confidence in their ability to perform it themselves and required the assistance of a healthcare professional. Others were concerned about contaminating the specimen if they took the test at home, so they preferred to have it done at a healthcare facility. However, some women preferred to take the test at home because it was more convenient and private. Another option available for self-sampling screening involves a community-based approach, it has been successfully implemented in cervical cancer screen-and-treat programmes in some countries within the SSA region [82]. This approach can be implemented by leveraging the presence of community health workers who have a long-standing relationship with the community. Further research must be conducted to establish acceptable and feasible delivery approaches for HPV self-sampling-based screening to match the needs of the end users. Increased screening uptake has been observed when services are offered for free. The majority of women who do not attend or lack access to screening services are from low socio-economic backgrounds, and offering free screening and treatment services can promote the uptake of available services. The governments and international partners working in cervical cancer prevention and control can forge strong partnerships to deliver free screening and treatment services to the women in the SSA region. One way to do this is to integrate HPV testing via self-sampling into existing sexual and reproductive health services already offered to women in health facilities.

To ensure that self-sampling has a public health impact, women who test positive for HPV should be triaged with another method, such as VIAC, to determine eligibility for treatment. The failure to access treatment services after screening has been identified as a barrier to self-sampling screening uptake. To ensure the success of the intervention, programme managers should ensure quick turnaround times for HPV tests by using low-cost point-of-care (POC) testing platforms such as GeneXpert, which produces results in an hour, allowing for same-day screen and treat or screen, triage and treat approaches to minimise loss to follow-up [83]. It is also critical to notify clients of positive test results immediately without delay using mobile communication technologies such as the short message service (SMS) system where there is available telecommunications infrastructure.

### 6.1 Strengths and limitations

The strength of this study lies in the systematic search of the literature in multiple databases. to identify relevant qualitative studies that met the inclusion criteria. We utilised the SEM to accurately identify the factors influencing the acceptability and uptake of HPV self-sampling. Our methodological quality assessment was conducted using the CASP tool to enhance the quality and confidence of our findings. Another strength of this study was the use of independent reviewers for the different stages of the systematic review. To minimise researcher subjectivity, we ensured that standard guidelines for conducting a systematic review were followed and that inductive doing was used during thematic analysis to avoid introducing any preconceived researcher ideas or notions. It is essential to note that although experiences and perspectives on self-sampling may vary across different societies and cultural contexts, our study provides valuable insights into the most common drivers and deterrents of self-sampling in SSA. Our findings are based on more than half of the studies that utilised focus group discussions as a data collection method; we acknowledge that focus groups may result in participants holding back important information. However, we remain confident in our findings as we followed established guidelines for systematic reviews to ensure transparency and minimise potential biases during study selection, extraction, and synthesis. It is important to note that the limitations associated with the review may influence the findings of this study; however, we believe the analysis was transparent and robust and that these findings are important for informing practice and future research.

## 7 Recommendations for future research

**Fig. 2:**
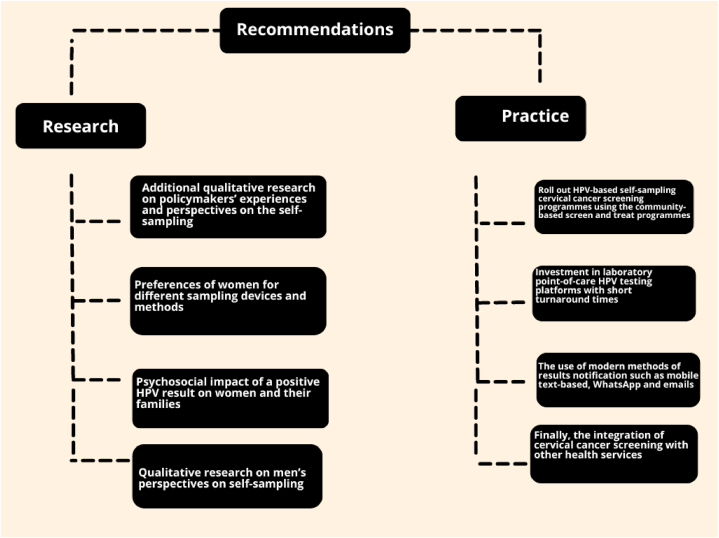
Recommendations for future research and practice.

Self-sampling for cervical cancer screening is an acceptable method, according to recent studies. We have made recommendations (Fig. 2) for future research and practice based on our findings. Additional qualitative research is needed to understand the perspectives of policymakers, program managers, and women's preferences for sampling methods. There is a need for additional qualitative research on the psychosocial impact of a positive HPV result and qualitative research to determine the perspectives of male partners and community leaders on cervical cancer screening, including self-care interventions such as HPV self-sampling. It is also important to conduct further research to establish women's preferences for delivering an HPV self-sampling screening intervention.

### 7.1 Implications for practice

The use of POC HPV testing platforms can ensure quicker turnaround times, making same-day screening and treatment feasible to prevent the loss of women to follow-up. Policymakers should also invest in modern communication methods such as mobile text-based notifications for easier notification of results. Additionally, integrating HPV self-sampling screening with other sexual reproductive health services for women may encourage screening uptake and also ensure cost-cutting for the health system due to the sharing of infrastructure and human resources. To ensure the buy-in of the community, including men, there is a need for robust community engagement tailored for men to improve their understanding of cervical cancer screening and other female reproductive health issues. A practical way to implement this is to visit men in areas they frequent, such as social clubs.

## 8 Conclusion

An interplay of individual, interpersonal, community and health system factors influence the acceptability and uptake of an HPV-based self-sampling intervention for cervical cancer screening. Through the insights of key stakeholders, we gained a deeper understanding of the key drivers and deterrents of an HPV-based self-sampling intervention. Irrespective of the strong evidence of HPV self-sampling acceptability, the lack of self-efficacy was a common concern, and women also expressed the need for early notification of their HPV results and the provision of treatment services after a positive HPV test. Addressing the reported barriers to self-sampling uptake by women is one way to ensure the success of future interventions in SSA. Currently, there are no standardised optimal delivery approaches for HPV self-sampling in SSA, and this calls for policymakers and programme managers to conduct research to decide on the most effective and acceptable approaches before implementation. HPV-based self-sampling for cervical cancer screening is a useful innovation with the potential to propel countries to attain WHO global elimination targets for cervical cancer by the end of the century.

## Funding

The authors received no funding from any organisation for this study.

## Ethics approval and consent to participate

Not applicable.

## Consent for publication

Not applicable.

## Data availability

Has data associated with your study been deposited into a publicly available repository?

No, but all data generated or analysed during this study are included in this published article or as supplementary materials.

## CRediT authorship contribution statement

**Mathias Dzobo:** Writing – review & editing, Writing – original draft, Methodology, Formal analysis, Data curation, Conceptualization. **Tafadzwa Dzinamarira:** Writing – review & editing, Writing – original draft, Supervision, Methodology, Conceptualization. **Ziningi Jaya:** Writing – review & editing, Methodology. **Kabelo Kgarosi:** Software, Methodology. **Tivani Mashamba-Thompson:** Writing – review & editing, Writing – original draft, Supervision, Methodology, Conceptualization.

## Declaration of generative AI and AI-assisted technologies in the writing process

During the preparation of this work, the author(s) used Grammarly to improve grammar use. After using this tool/service, the author(s) reviewed and edited the content as needed and take(s) full responsibility for the content of the publication.

## Declaration of competing interest

The authors declare that they have no known competing financial interests or personal relationships that could have appeared to influence the work reported in this paper.
