# Supplementary material for: Experiences and perspectives regarding human papillomavirus self-sampling in sub-Saharan Africa: A systematic review of qualitative evidence
Source: Heliyon. 2024 Jun 18;10(12):e32926. doi: 10.1016/j.heliyon.2024.e32926 (PMC11253232; doi:10.1016/j.heliyon.2024.e32926)
Supplement: Multimedia component 1 [file mmc1.docx]

**SUPPLEMENTARY FILES**

**Supplementary File 1:** Database Search Results

| **Date of search** | **Electronic Database** | **Keywords/MeSH terms** | **Number of retrieved studies** |
| --- | --- | --- | --- |
| 14-06-2021 | Web of Science | Human papillomavirus*" OR alphapapillomavirus OR hpv OR papillomavirus* OR "Cervical cancer*" OR "Uterine Cervical Neoplasm*" OR "cancer of the cervix" OR "uterine cervix tumor" AND "self-sampling" OR "self sampl*" OR "self collect*" OR "self screen*" OR screening AND Africa OR "sub-Saharan Africa" OR "Africa South of the Sahara" AND female OR woman OR women NOT algeria OR egypt OR libya OR morocco OR tunisia | 533 |
|  |  |  |  |
| 06-07-2021 | PubMed | ((("Uterine Cervical Neoplasms"[Mesh] OR "Uterine Cervical Neoplasm*"[tw] OR "Cervical Cancer"[tw] AND (female[Filter])) OR ("Alphapapillomavirus"[Mesh] OR Alphapapillomavirus[tw] OR "Human papillomavirus*"[tw] OR HPV[tw] OR papillomavirus*[tw] AND (female[Filter]))) AND ("Self Administration"[Mesh] OR self-sampl*[tw] OR "self collect*"[tw] OR "self Administ*"[tw] OR "self screen*"[tw] AND (female[Filter]))) AND ("Africa South of the Sahara"[Mesh] OR "Africa Sub-Saharan"[tw] OR "Subsaharan Africa"[tw] OR "Sub-Sahara africa"[tw] AND (female[Filter])) Filters: in the last 10 years, Female | 122 |
|  |  |  |  |
| 06-07-2021 | Scopus | (TITLE-ABS-KEY(Africa* OR "sub-Saharan Africa" OR SS OR "Africa South of the Sahara" OR  "Subsahara* Africa") AND TITLE-ABS-KEY("Human papillomavirus*" OR alphapapillomavirus OR  hpv OR papillomavirus* OR "Cervical cancer*" OR "Uterine Cervical Neoplasm*" OR "cancer of  the cervix" OR "uterine cervix tumor") AND TITLE-ABS-KEY("self-sampling" OR "self sampl*" OR  "self collect*" OR "self screen*" OR screening) AND NOT TITLE-ABS-KEY(Algeria OR Egypt OR  Libya OR Morocco OR Tunisia)) AND ( LIMIT-TO ( PUBYEAR,2021) OR LIMIT-TO (  PUBYEAR,2020) OR LIMIT-TO ( PUBYEAR,2019) OR LIMIT-TO ( PUBYEAR,2018) OR LIMIT-TO (  PUBYEAR,2017) OR LIMIT-TO ( PUBYEAR,2016) OR LIMIT-TO ( PUBYEAR,2015) OR LIMIT-TO (  PUBYEAR,2014) OR LIMIT-TO ( PUBYEAR,2013) OR LIMIT-TO ( PUBYEAR,2012) OR LIMIT-TO (  PUBYEAR,2011) ) AND ( LIMIT-TO ( SRCTYPE,"j" ) ) | 224 |
| 12-07- 2021 | Ovid Medline | Ovid MEDLINE(R) <1996 to August Week 3 2021>  1 exp Uterine Cervical Neoplasms/ 48683  2 Uterine Cervical Neoplasms.af. 48719  3 Cervical Cancer.af. 37377  4 exp Alphapapillomavirus/ 8338  5 Alphapapillomavirus.af. 2733  6 exp Papillomavirus Infections/ 31367  7 Papillomavirus Infection*.af. 28075  8 Human papillomavirus*.af. 33426  9 HPV.af. 35627  10 papillomavirus*.af. 42280  11 Cervi* Cancer.af. 38169  12 1 or 2 or 3 or 4 or 5 or 6 or 7 or 8 or 9 or 10 or 11 86646  13 exp Self Administration/ 8841  14 Self Administration.af. 11764  15 self-sampl*.af. 682  16 self collect*.af. 1155  17 self administrat*.af. 12471  18 self screen*.af. 205  19 self-testing.af. 1129  20 self-test*.af. 1491  21 13 or 14 or 15 or 16 or 17 or 18 or 19 or 20 15594  22 exp "Africa South of the Sahara"/ 168852  23 sub-sahara* Africa.af. 19913  24 22 or 23 173452  25 12 and 21 and 24 101  26 limit 25 to yr="2011 -Current" 95  27 limit 26 to (female and humans) 95 | 95 |
| 14-07-2021 | Cochrane | ID Search Hits  #1 MeSH descriptor: [Alphapapillomavirus] explode all trees 247  #2 MeSH descriptor: [Uterine Cervical Neoplasms] explode all trees 2171  #3 ("Uterine Cervical Neoplasm*" OR "Cervical Cancer" OR Alphapapillomavirus OR "Human papillomavirus*" OR HPV OR papillomavirus*) (Word variations have been searched) 7022  #4 #1 OR #2 OR #3 6989  #5 MeSH descriptor: [Self Administration] explode all trees 778  #6 (self-sampl* OR "self collect*" OR "self Administ*" OR "self screen*") (Word variations have been searched) 6495  #7 #5 OR #6 1040  #8 MeSH descriptor: [Africa South of the Sahara] explode all trees 6811  #9 ("sub-Saharan Africa") (Word variations have been searched) 2037  #10 #8 OR #9 8279  #11 #4 AND #7 AND #10 8  Date range 2011-2021 Results 7 | 7 |
| 31-08-21 | Grey Literature identified through other sources | “Human papillomavirus” OR “cervical cancer” AND “self-sampling” AND “sub-Saharan Africa” | 37 |
|  | Total articles |  | 1018 |

**Extended search strategy from July 2021-March 2024**

| **Date of search** | **Electronic Database** | **Keywords/MeSH terms** | **Number of retrieved studies** |
| --- | --- | --- | --- |
| 14-03-2023 | Web of Science | Human papillomavirus*" OR alphapapillomavirus OR hpv OR papillomavirus* OR "Cervical cancer*" OR "Uterine Cervical Neoplasm*" OR "cancer of the cervix" OR "uterine cervix tumor" AND "self-sampling" OR "self sampl*" OR "self collect*" OR "self screen*" OR screening AND Africa OR "sub-Saharan Africa" OR "Africa South of the Sahara" AND female OR woman OR women NOT algeria OR egypt OR libya OR morocco OR tunisia | 213 |
|  |  |  |  |
| 14-03-2023 | PubMed | ((("Uterine Cervical Neoplasms"[Mesh] OR "Uterine Cervical Neoplasm*"[tw] OR "Cervical Cancer"[tw] AND (female[Filter])) OR ("Alphapapillomavirus"[Mesh] OR Alphapapillomavirus[tw] OR "Human papillomavirus*"[tw] OR HPV[tw] OR papillomavirus*[tw] AND (female[Filter]))) AND ("Self Administration"[Mesh] OR self-sampl*[tw] OR "self collect*"[tw] OR "self Administ*"[tw] OR "self screen*"[tw] AND (female[Filter]))) AND ("Africa South of the Sahara"[Mesh] OR "Africa Sub-Saharan"[tw] OR "Subsaharan Africa"[tw] OR "Sub-Sahara africa"[tw] AND (female[Filter])) | 44 |
|  |  |  |  |
| 14-03-2023 | Scopus | (TITLE-ABS-KEY(Africa* OR "sub-Saharan Africa" OR SS OR "Africa South of the Sahara" OR  "Subsahara* Africa") AND TITLE-ABS-KEY("Human papillomavirus*" OR alphapapillomavirus OR  hpv OR papillomavirus* OR "Cervical cancer*" OR "Uterine Cervical Neoplasm*" OR "cancer of  the cervix" OR "uterine cervix tumor") AND TITLE-ABS-KEY("self-sampling" OR "self sampl*" OR  "self collect*" OR "self screen*" OR screening) AND NOT TITLE-ABS-KEY(Algeria OR Egypt OR  Libya OR Morocco OR Tunisia)) AND ( LIMIT-TO ( PUBYEAR,2021) OR LIMIT-TO (  PUBYEAR,2020) OR LIMIT-TO ( PUBYEAR,2019) OR LIMIT-TO ( PUBYEAR,2018) OR LIMIT-TO (  PUBYEAR,2017) OR LIMIT-TO ( PUBYEAR,2016) OR LIMIT-TO ( PUBYEAR,2015) OR LIMIT-TO (  PUBYEAR,2014) OR LIMIT-TO ( PUBYEAR,2013) OR LIMIT-TO ( PUBYEAR,2012) OR LIMIT-TO (  PUBYEAR,2011) ) AND ( LIMIT-TO ( SRCTYPE,"j" ) ) | 145 |
| 14-03-2023 | Ovid Medline | Ovid MEDLINE(R) <1996 to August Week 3 2021>  1 exp Uterine Cervical Neoplasms/ 48683  2 Uterine Cervical Neoplasms.af. 48719  3 Cervical Cancer.af. 37377  4 exp Alphapapillomavirus/ 8338  5 Alphapapillomavirus.af. 2733  6 exp Papillomavirus Infections/ 31367  7 Papillomavirus Infection*.af. 28075  8 Human papillomavirus*.af. 33426  9 HPV.af. 35627  10 papillomavirus*.af. 42280  11 Cervi* Cancer.af. 38169  12 1 or 2 or 3 or 4 or 5 or 6 or 7 or 8 or 9 or 10 or 11 86646  13 exp Self Administration/ 8841  14 Self Administration.af. 11764  15 self-sampl*.af. 682  16 self collect*.af. 1155  17 self administrat*.af. 12471  18 self screen*.af. 205  19 self-testing.af. 1129  20 self-test*.af. 1491  21 13 or 14 or 15 or 16 or 17 or 18 or 19 or 20 15594  22 exp "Africa South of the Sahara"/ 168852  23 sub-sahara* Africa.af. 19913  24 22 or 23 173452  25 12 and 21 and 24 101  26 limit 25 to yr="2011 -Current" 95  27 limit 26 to (female and humans) 95 | 47 |
| 14-03-2023 | Cochrane | ID Search Hits  #1 MeSH descriptor: [Alphapapillomavirus] explode all trees 247  #2 MeSH descriptor: [Uterine Cervical Neoplasms] explode all trees 2171  #3 ("Uterine Cervical Neoplasm*" OR "Cervical Cancer" OR Alphapapillomavirus OR "Human papillomavirus*" OR HPV OR papillomavirus*) (Word variations have been searched) 7022  #4 #1 OR #2 OR #3 6989  #5 MeSH descriptor: [Self Administration] explode all trees 778  #6 (self-sampl* OR "self collect*" OR "self Administ*" OR "self screen*") (Word variations have been searched) 6495  #7 #5 OR #6 1040  #8 MeSH descriptor: [Africa South of the Sahara] explode all trees 6811  #9 ("sub-Saharan Africa") (Word variations have been searched) 2037  #10 #8 OR #9 8279  #11 #4 AND #7 AND #10 | 2 |
|  | Total articles |  | 451 |

**Supplementary File 2:** Critical Appraisal of Included studies

| **Author & Year** | **1** | **2** | **3** | **4** | **5** | **6** | **7** | **8** | **9** | **10** | **Methodological limitation** |
| --- | --- | --- | --- | --- | --- | --- | --- | --- | --- | --- | --- |
| Saidu et al, 2019 | Y | Y | Y | Y | Y | C | Y | Y | Y | Y | NO |
| Bakiewicz et al, 2020 | Y | Y | C | Y | Y | N | Y | Y | Y | Y | Minor |
| Lee et al, 2021 | Y | Y | Y | Y | Y | N | Y | Y | Y | Y | Minor |
| Oketch et al, 2019 | Y | Y | Y | Y | Y | C | Y | Y | Y | Y | NO |
| Behnke et al, 2020 | Y | Y | Y | Y | Y | N | Y | Y | Y | Y | Minor |
| Brandt et al, 2019 | Y | Y | Y | Y | Y | Y | Y | Y | Y | Y | NO |
| Bansil et al, 2014 | Y | Y | Y | Y | Y | C | Y | Y | Y | Y | NO |
| Roux et al, 2021 | Y | Y | Y | Y | Y | C | Y | C | Y | Y | Minor |
| Rawat et al, 2021 | Y | Y | Y | Y | Y | C | Y | Y | Y | Y | NO |
| Mensah et al, 2020 | Y | Y | Y | Y | Y | C | Y | Y | Y | Y | NO |
| Megersa et al, 2020 | Y | Y | Y | Y | Y | C | Y | Y | Y | Y | NO |
| Podolak et al, 2017 | Y | Y | Y | C | C | Y | C | Y | Y | Y | Minor |
| Teng et al, 2014 | Y | Y | Y | Y | Y | C | Y | Y | Y | Y | NO |

**KEY**

Y=YES, N=NO, C= can’t tell, Q=Question

*Q1. Was there a clear statement of the aims of the research?*

*Q2. Is a qualitative methodology appropriate?*

*Q3. Was the research design appropriate to address the aims of the research?*

*Q4. Was the recruitment strategy appropriate to the aims of the research?*

*Q5. Was the data collected in a way that addressed the research issue?*

*Q6. Has the relationship between researcher and participants been adequately considered?*

*Q7. Have ethical issues been taken into consideration?*

*Q8. Was the data analysis sufficiently rigorous?*

*Q9. Is there a clear statement of findings?*

*Q10. How valuable is the research?*
